# Immune responses to the 105AD7 human anti-idiotypic vaccine after intensive chemotherapy, for osteosarcoma

**DOI:** 10.1038/sj.bjc.6602500

**Published:** 2005-03-29

**Authors:** K Pritchard-Jones, I Spendlove, C Wilton, J Whelan, S Weeden, I Lewis, J Hale, C Douglas, C Pagonis, B Campbell, P Alvarez, G Halbert, L G Durrant

**Affiliations:** 1 Royal Marsden Hospital and Institute of Cancer Research, Downs Road, Sutton, Surrey SM2 5PT, UK; 2 The Department of Clinical Oncology, The University of Nottingham, City Hospital, Nottingham NG5 1PB, UK; 3 Middlesex Hospital, Nassau Street, London W1T 3AA, UK; 4 Cancer Division, MRC Clinical Trials Unit, 222 Euston Road, London NW1 2DA, UK; 5 St James's University Hospital, Beckett Street, Leeds, West Yorkshire LS9 7TF, UK; 6 Newcastle General Hospital, Westgate Road, Newcastle-upon-Tyne NE4 6BE, UK; 7 UKCCSG, University of Leicester, 9 Princess Road West, Leicester LE1 6TH, UK; 8 Cancer Research UK Drug Development Office, 61 Lincoln's Inn Fields, London WC2A 3PX, UK; 9 Cancer Research UK Formulation Unit, Department of Pharmaceutical Sciences, Royal College Building, University of Strathclyde, 204 George Street, Glasgow G1 1XW, UK

**Keywords:** cancer vaccines, osteosarcoma, clinical trial, immune responses, anti-idiotypic antibodies

## Abstract

105AD7 is a human monoclonal antibody that mimics the complement regulatory protein, CD55, overexpressed by many solid tumours including osteosarcoma. This study was designed to assess the toxicity and efficacy of this vaccine in a young age group of patients within 1–6 months of myleosuppressive chemotherapy. Out of 28, 20 (71%, 95% CI 51–87%) patients showed a significant T-cell proliferation response *in vitro* to the 105AD7 protein but not to human IgG. Furthermore, 13 out of 22 (59%, 95% CI 36–79%) patients showed antigen-specific *γ*IFN secretion (range 20–370 U/ml). Nine out of 28 (32%, 95% CI 16–52%) patients made weak antibody responses to CD55. This study showed that 105AD7 was well tolerated in younger patients with osteosarcoma. In addition, two patients with possible clinical responses were given compassionate permission to continue immunisation quarterly for 2 years. They both remain alive and disease free 5.8 and 6.5 years from original diagnosis of osteosarcoma and showed no adverse effects of repeated immunisation. In conclusion, the majority of patients showed measurable T helper responses when vaccination was commenced within a 6-month window of intensive chemotherapy with no clinically significant toxicity. Future clinical trials incorporating immune stimulation strategies should include early introduction of vaccines during the highest risk period for relapse.

Osteosarcoma is a high-grade bone tumour that affects mainly adolescents and young adults. Two-thirds of patients diagnosed before the age of 30 years have a localised primary tumour in a limb bone. Of these, about 50–60% can expect to be long-term disease-free survivors with modern therapies that include intensive multiagent chemotherapy and surgical resection (Souhami *et al*, 1997; Fuchs *et al*, 1998). The remaining one-third of patients with osteosarcoma have a poorer prognosis and present with either a primary in the axial skeleton or metastatic disease. Even among localised disease, most relapses occur within 2 years of initial diagnosis, and a third of patients relapse or die within 6 months of the scheduled end of multiagent chemotherapy. Comparisons of the several national and international clinical trials of manipulation of chemotherapy type and dose intensity suggest a limited role for conventional chemotherapy in further improvements in disease control. Hence, there is a need to search for novel approaches.

A possible therapeutic role for immunomodulation in osteosarcoma was suggested by the naturally occurring dog model of this disease, in which injections of the macrophage activator, liposomal encapsulated muramyl tripeptide phosphatidyl ethanolamine (MTP-PE) induced regression. The role of MTP-PE as an immune stimulatory adjuvant therapy was tested in a recent trial conducted by the Children's Oncology Group in North America. This 2 × 2 factorial trial randomised patients to receive control chemotherapy (methotrexate, doxorubicin and cisplatin) with or without the addition of ifosfamide and with or without MTP-PE. Preliminary results of this trial have indicated that although neither treatment offers an event-free survival benefit individually, there is a synergistic effect when ifosfamide and MTP-PE are administered together (Meyers *et al*, 2001) Although suggestive, this study does not provide conclusive evidence of the benefit from this form of immunomodulation.

In order to impact on the early relapses, any immunotherapy approach would have to be commenced relatively early in the course of treatment. Since the chemotherapy given for osteosarcoma is very myelosuppressive, we wished to test whether patients could mount an immune response to a relevant tumour antigen if a vaccination strategy was commenced within 1–6 months of completion of chemotherapy. We chose to use an anti-idiotypic monoclonal antibody, 105AD7, whose safety had been demonstrated in two previous trials in patients with colorectal cancer.

105AD7 is a human anti-idiotypic monoclonal antibody that was cloned from a cancer patient who had received the radiolabelled mouse monoclonal 791T/36 for diagnostic imaging (Austin *et al*, 1989). 791T/36 was subsequently shown to recognise CD55, a complement regulatory protein overexpressed by tumours to protect them from complement attack. 105AD7 was shown to have both amino acid and structural homology with CD55 and could therefore be used as a vaccine to induce antitumour immune responses (Spendlove *et al*, 2000).

In a phase I clinical trial in metastatic colorectal cancer patients, 105AD7 induced antitumour inflammatory responses with no associated toxicity (Denton *et al*, 1994). However, in a subsequent double-blind randomised phase II trial there was no survival benefit in the setting of measurable disease (Maxwell-Armstrong *et al*, 2001). This may have been due to the immunosuppressive effects of large tumour burden as a neoadjuvant trial showed promising results indicating a valuable immune response (Durrant *et al*, 2000a, 2000b; Schwann *et al*, 2000). As 791T/36 was originally raised against a human osteogenic tumour cell line and was used successfully to image osteosarcomas *in vivo* (Farrands *et al*, 1983), we proposed that 105AD7 may be a suitable vaccine for treatment of this disease. Previous experience with colorectal cancer suggested that 105AD7 would be most effective in treatment of patients with minimal residual disease.

This study had two main aims: to assess the toxicity of vaccinating osteosarcoma patients with 105AD7 and to determine the ability of these patients to raise an immune response to this vaccine.

## MATERIALS AND METHODS

### Patients

Patients were recruited from four centres in England (The Middlesex Hospital, London; Royal Marsden Hospital, Sutton, Royal Victoria Infirmary, Newcastle and St James Hospital, Leeds). Eligible patients had a diagnosis of high-grade osteosarcoma, were aged <30 years at time of study entry and had a lymphocyte count of >0.5 × 10^9^ ml^−1^, and biochemical indices of renal and liver function within three times the age-related upper limit of normal.

Patients were recruited if they were deemed at high risk of treatment failure because they had presented with metastatic disease, had experienced at least one relapse or if they presented with a localised limb primary tumour and who were not participating in another clinical trial. Patients who generally have a better outlook were included in this phase I/II study due to the good safety profile of the vaccine in previous studies in adult colorectal cancer patients. All patients had to have completed current best standard therapy for their situation and to have received their last dose of chemotherapy within the previous 1–6 months. All patients had a Lansky performance score of >80 or a Karnofsky performance score of >60%, according to age. Exclusion criteria were any autoimmune disease, lymphocyte count <0.5 × 10^9^ l^−1^, platelet count 50 × 10^9^ l^−1^, concomitant anticancer treatment or other vaccinations within the previous 3 weeks. All patients (and their parents when the patient was aged less than 16 years) gave fully informed, written consent to participate in the study.

Patients were required to have baseline imaging studies, appropriate to their clinical situation and follow-up, immediately prior to study entry, to confirm either clinical remission or presence of disease. Patients with measurable disease were required to have the appropriate imaging modality repeated at week 9, to look for response, and again at week 14, to assess durability of response.

### Vaccination schedule and venous blood sample handling

**Figure 1:**
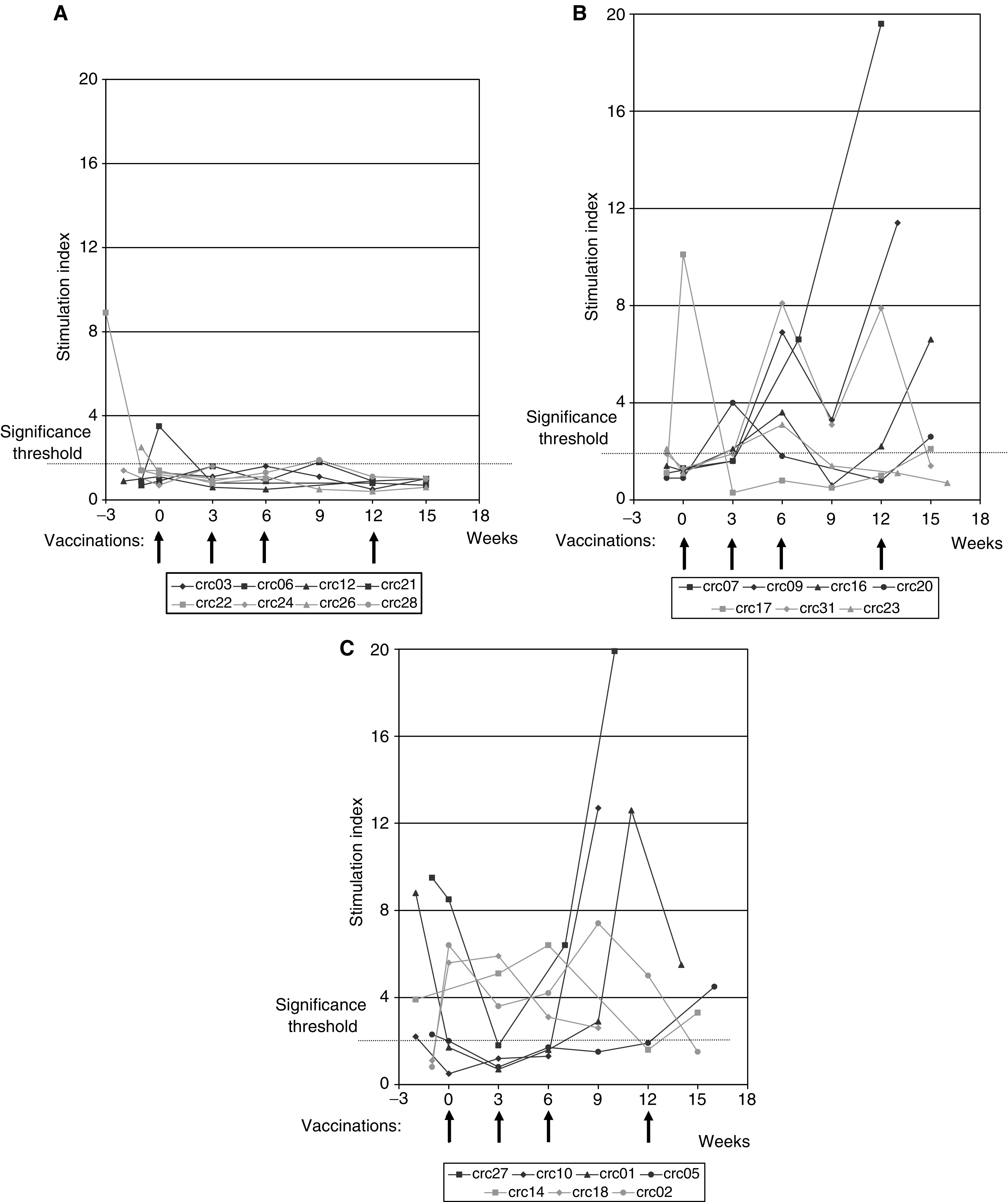
(**A**) Patients showing no proliferative response to 105AD7 or human IgG control. (**B**) Patients showing proliferative response to 105AD7 but not human IgG control (no prevaccination response). (**C**) Patients showing proliferative response to 105AD7 but not human IgG control (with prevaccination response). T-cell proliferation was assessed by ^3^H-labelled thymidine incorporation following 5-day stimulation with either 105AD7 or control human IgG: 
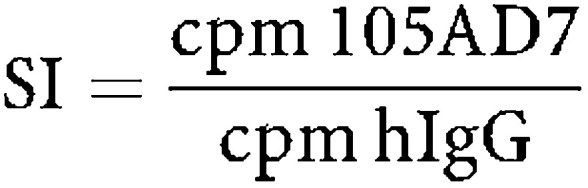 An SI of greater than 2 is considered significant. Arrows denote injection with 105AD7 by intrademal (10 *μ*g) and intramuscular (100 *μ*g on alum) injection.

The vaccination schedule and blood sampling are shown in Figure 1. For isolation of lymphocytes, a 20 ml baseline venous blood sample was taken in preservative-free heparin during the week prior to commencement of vaccination and again at week 0, 3, 6, 9, 12 and 15. Full blood count, urea, electrolytes, creatinine and liver function tests were performed at weeks 0, 3, 6 and 12 at the clinical trial centre for that patient. Patients received an intradermal (i.d.) injection of 10 *μ*g of 105AD7 and an intramuscular (i.m.) dose of 100 *μ*g of 105AD7 adsorbed on alyhdrogel 85 at weeks 0, 3, 6 and 12, immediately following the blood sampling. The venous blood samples were sent by overnight courier to the immune analysis laboratory where lymphocytes were separated on Lymphoprep (Flow Laboratories, Irvine, Scotland) and stored at −180°C. Plasma was also stored at −80°C.

This trial was carried out under the auspices of the Cancer Research UK Drug Development Office with local ethical approval in all centres. The study was carried out in accordance with the Declaration of Helsinki 1996.

### Antibody

Clinical grade 105AD7 human antibody was produced as described previously using the guidelines of the Cancer Research Campaign, United Kingdom (Denton *et al*, 1994). The trial supplies were prepared under cGMP conditions. Antibody for immunisation was prepared adsorbed on alhydrogel 85 (Superfos Biosector now Brenntag Biosector A/S, Elsenbakken, Denmark) at 100 *μ*g ml^−1^ in sterile, pyrogen-free phosphate-buffered saline in a 1 ml type 1 glass ampoule containing 1 ml. The weight ratio of 105AD7 to alhydrogel 85 was 1 : 10. The skin test dose was prepared at 100 *μ*g ml^−1^ in sterile, pyrogen-free phosphate-buffered saline in a 1-ml type 1 glass ampoule containing 0.125 ml. Stability studies have shown that the antibody can be stored at 4°C for a minimum of 5 years with no loss in binding activity.

### T-cell analysis

A complete set of cryopreserved lymphocytes for each patient was defrosted for simultaneous analysis. A sample of peripheral blood mononuclear cells (PBMCs) was removed, irradiated and used as antigen-presenting cells. The remaining PBMCs were stimulated with these antigen-presenting cells in the presence of 105AD7 (10 *μ*g ml^−1^) or control human IgG antibody (10 *μ*g ml^−1^) in RPMI (Flow laboratories) containing 10% allogeneic human serum at 10^6^ cell ml^−1^ in 24-well plates. All cultures were incubated in a humidified incubator of 5% CO_2_ and 95% air at 37°C. Proliferation of lymphocytes was quantified after 5 days by resuspending the cells and removing four 100 *μ*l aliquots and incubating these cells in fresh 96-well plates overnight in the presence of 3[H]thymidine. All experiments were in quadruplicate and there was always an s.d. of less than 10%. Each time course was repeated twice and if there was any discordance it was repeated a third time. The response to human IgG was not significantly different from the medium control by a one-way analysis of variance. For clarity, results on figures and tables are presented as a stimulation index (SI)=incorporated counts per minute (cpm) in the presence of 105AD7/cpm in the presence of Human IgG. One-way analysis of variance showed that all samples that responded with a two-fold increase in response to 105AD7 as compared to human IgG were significant.

### *γ*IFN Secretion

*γ*IFN secretion was analysed in tissue culture supernatant of lymphocytes that had been stimulated with either 105AD7 (10 *μ*g ml^−1^) or human IgG (10 *μ*g ml^−1^) for 5 days *in vitro γ*IFN was detected by ELISA (R&D, Abingdon, Oxon, UK).

### Antibody response

CD55 and CEA were purified from 791Tcells and colorectal tumours using previously described methods (Price *et al*, 1987; Spendlove *et al*, 1999) and used to coat microtitre plates by overnight incubation at room temperature. Plates were blocked with phosphate buffer and saline (PBS) containing 1% bovine serum albumin (BSA). Plasma samples were defrosted, diluted in PBS containing 0.1% BSA and incubated with the antigens for 1 h at 4°C. Bound antibodies were detected by rabbit anti-human horseradish peroxidase (Dako, Denmark) and TMB substrate (R and D). Absorbance was read at 402 mm on a plate reader.

### HLA typing

Patients MHC class II phenotyping was measured by standard molecular techniques.

### Statistical methodology

Due to the small number of patients available to the study and the fact that this vaccine had not previously been tested in osteosarcoma, the sample size was determined using the single stage procedure proposed by Fleming (1982). This method makes use of the response probabilities of interest, and allows statistically meaningful designs to be constructed with relatively small patient numbers. Sample sizes for Fleming's single stage procedure are tabulated by A’Hern (2001). The lowest response probability of interest is set as 0.50 and the highest is set as 0.75. To reliably evaluate the immunological response rate for the new vaccine (significance level=5%, power=80%), 23 patients would have to be entered into the study.

Statistical analysis was carried out using SPSS Version 1 for Windows. Exact 95% confidence intervals (CI) for response rates were obtained from Geigy Scientific Tables (Altman *et al*, 2000).

## RESULTS

This trial had two main aims. The phase I component was to assess the toxicity profile of this agent in children and young adults, since the previous excellent safety profile had been documented only in older adults with colorectal cancer. The phase II component addressed the question of whether 105AD7 was capable of stimulating a specific immune response in this relatively heavily pretreated group of patients within 1–6 months of their last dose of chemotherapy. The evaluation of any clinical responses where possible was as a secondary end point

In total, 31 patients were recruited to the study from August 1998 to January 2001. One withdrew prior to receiving any vaccine as a baseline CT scan showed recurrent disease. Two further patients withdrew after one or two doses of vaccine due to early progressive disease. The remaining 28 patients received three (5) or four (23) doses of vaccine. In total, 30 patients were evaluated for toxicity, having received at least one dose of vaccine, and 28 had received at least three doses of vaccine to give sufficient time points of blood sampling for analysis of immune response.

### Immune responses

The immune response of patients to the anti-idiotypic vaccine was assayed by *in vitro* T-cell proliferation responses following exposure to 105AD7 and to control human IgG. In a subset of patients, stimulation of secretion of *γ*IFN following the same stimulator was also measured. Overall 20 out of 28 (71%, 95% CI 51–87%) patients showed a proliferative response to 105AD7 but not to human Ig. Patients’ proliferation responses are summarised in Table 1. Patients PBMC proliferative responses could be divided into three groups: Group I (eight patients) failed to show any significant proliferation response to 105AD7 postvaccination (Figure 1A). Group II (seven patients) showed lymphocyte proliferation in response to the vaccine but had no pre-existing response to 105AD7 (Figure 1B). Group III (13 patients) showed a pre-existing response to 105AD7, which was subsequently boosted by the vaccine (Figure 1C). Response data for all three groups are summarised in Table 1 and Figure 1.

In contrast to previous studies in chemonaïve colorectal cancer patients who responded to the first vaccination, a median of three doses of vaccine were required to observe a peak PBMC proliferative response in this group of patients with osteaosarcoma. A similar delayed response was observed in the group of patients who had evidence for a pre-existing response to 105AD7.

Previous studies have shown a correlation between response to 105AD7 and an expression of HLA-DR 1, 3, 7 phenotypes. Similar results were observed in this study with 11 out of 15 (73%) of patients expressing these phenotypes showing a proliferation response. However, this study suggests that patients expressing HLA-DR 13, 15 and 17 haplotypes may also respond to 105AD7 vaccine (Table 1).

In total, 22 patients were also assayed for secretion of γIFN. A total of 13 patients (59%, 95% CI 36–79%) secreted *γ*IFN in response to 105AD7 but not to pooled human immunoglobulin. There was a correlation between the proliferation assay and *γ*IFN secretion in 10 patients. Six patients showed a proliferation response but no significant secretion of *γ*IFN could be observed. Three patients showed *γ*IFN secretion to 105AD7 but failed to have a significant proliferation response. Three patients failed to respond in either assay.

**Figure 2:**
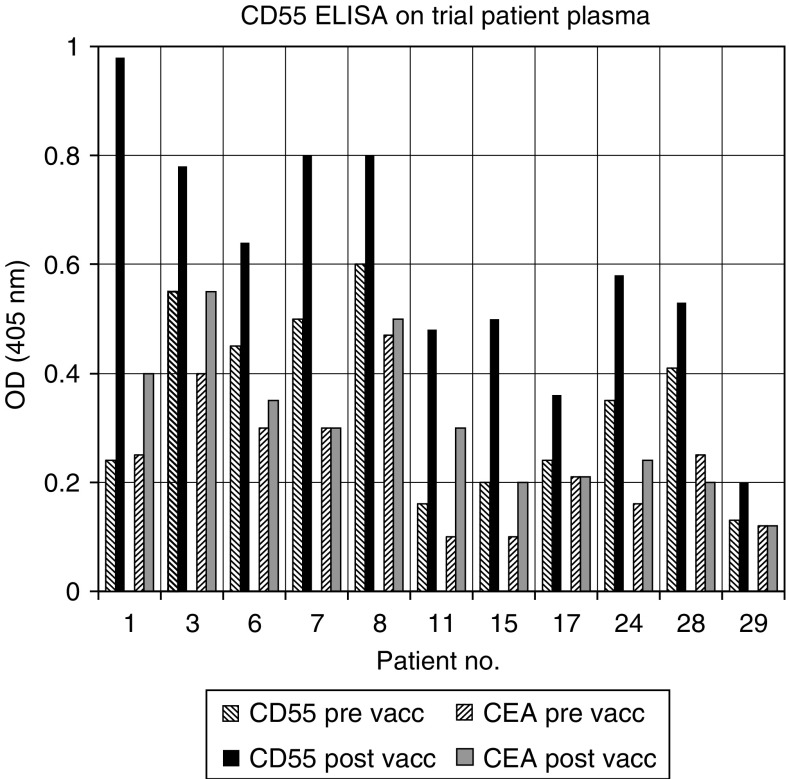
Antibody responses of patients to CD55 and control antigen CEA, pre- and postimmunisation with 105AD7. Antibodies were detected in patients serum by ELISA.

Patients were also screened for their ability to make an antibody response. The antigen 105AD7 mimics is CD55. At a dilution of 1/3, 11 out of 28 patients showed a boosted antibody response to CD55 that was greater than the response to CEA (carcinoembryonic antigen; Figure 2). A further six patients had a pre-existing response to CD55 that was not boosted by vaccination with 105AD7. All but one of these patients was in group III that also showed a pre-existing PBMC proliferative response to 105AD7 (data not shown). Of the nine patients showing an anti-CD55 response only five of these were titratable and two were IgM and three IgG responses.

### Toxicity

Of the 30 patients assessed for toxicity, 15 patients had a minor adverse event (grade 1 or 2) that was deemed probably or almost certainly related to administration of 105AD7. These events comprised 33 episodes of local symptoms at the injection site, lasting up to 24 h, and three episodes of intermittent chest pain in one patient and general lethargy in one other.

Two patients experienced exacerbation of tumour-related pain in the week following vaccination, one requiring hospitalisation for intravenous opiates. However, both of these patients had extensive disease at time of study entry, had experienced similar unstable pain prior to study entry and both had subsequent early progressive disease. One patient developed grade 3 lymphopaenia that resolved spontaneously after the second dose of vaccine and did not recur.

Two patients were given prolonged vaccination on compassionate grounds, as there was a possibility that they had had a benefit from the vaccine. They each continued both i.d. and i.m. vaccination on a 2–3 monthly basis until they were 2 years from the initial vaccination. Neither patient experienced any vaccine-related symptoms. This lack of toxicity is in keeping with previous clinical studies in colorectal cancer patients (Denton *et al*, 1994; Durrant *et al*, 2000a; Maxwell-Armstrong *et al*, 2001).

### Disease response

This study was not designed to test antitumour efficacy. Only five patients entered the study with measurable disease (Table 2). Three patients (CRC15, 22, 24) received only three doses of vaccine due to early disease progression, one patient (CRC7) completed all four doses as sole therapy for an early local recurrence of a pelvic primary tumour but progressed again shortly afterwards. The fifth patient (CRC9) showed evidence of possible response and remains well, as described below.

Table 2 shows the status at study entry and clinical course of all the patients in the trial, grouped according to their *in vitro* immune response to 105AD7. There were no obvious differences between the three immune response groups in either number of prior relapses, number with measurable disease, clinical risk group or median interval from end of last chemotherapy to study entry (18.5, 21 and 18 weeks for groups I, II and III respectively). In patients who failed to mount an immune response (group I) after 105AD7 vaccination, all those who showed evidence of disease subsequently died of disease. However, in those patients who demonstrated an immune response (Group II and III), there were five patients who remain alive and disease free (*n*=3, at 32, 49, 61 months from study entry) or alive with disease (*n*=2 at 34 and 50 months from study entry) although they showed evidence of disease.

**Figure 3:**
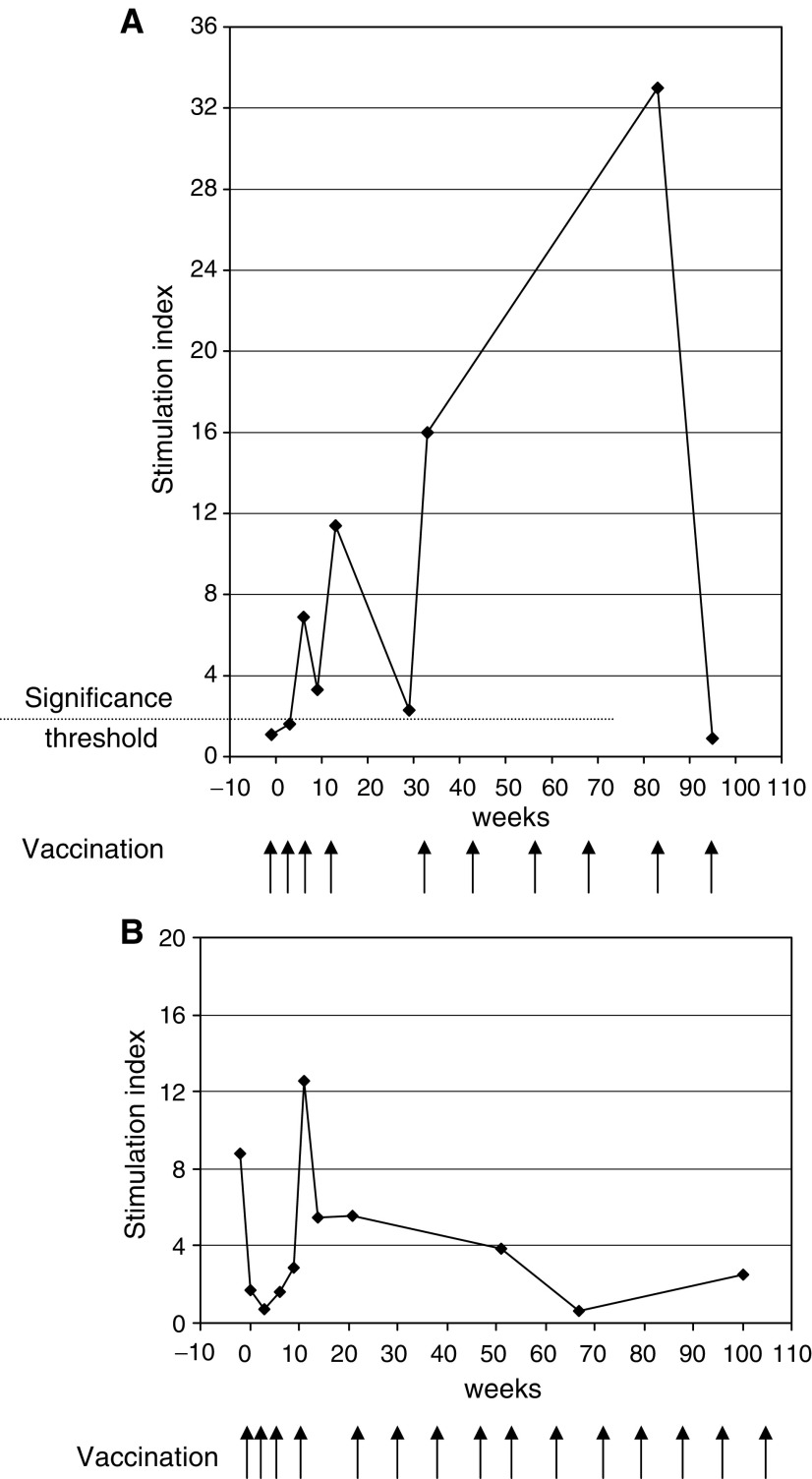
(**A**) Proliferation response of patient CRC09 following immunisation with 105AD7. (**B**) Proliferation response of patient CRC01 following immunisation with 105AD7. T-cell proliferation was assessed by ^3^H-labelled thymidine incorporation following 5-day stimulation with either 105AD7 or control human IgG: 
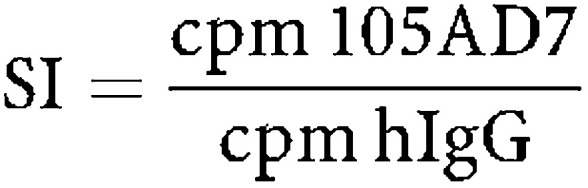 An SI of greater than 2 is considered significant. Arrows denote injection with 105AD7 by intradermal (10 *μ*g) and intramuscular (100 *μ*g on alum) injection.

Two patients showed unusual clinical courses CRC9 and CRC1. One CRC9 in response group II had presented 12 months prior to study entry with a pelvic primary and lung metastases that showed no response to conventional first line chemotherapy. She was therefore treated with an experimental approach combining high-dose chemotherapy and stem cell rescue with systemic intravenous radioactive samarium. Following completion of this, she was found to have residual inoperable lung metastases. As she had shown *in vitro* responses to 105AD7 (Figure 3A), she was allowed to continue on a compassionate basis with what was believed to be the best potential therapeutic vaccine schedule. CRC9 received a total of a further six doses of vaccine at 3 monthly intervals. Her *in vitro* immune response fell dramatically after the end of the formal study, suggesting that a memory response was not being established (Figure 3A). However, with resumption of vaccination this was rapidly boosted and reached a peak stimulation index of 32. Although this then fell to within the unmeasurable range on a single sample taken after completion of the prolonged vaccination, she remains free of signs of progressive disease 4.2 years from study entry and 2.2 years from her last dose of vaccine.

The second patient (CRC1) was in response group III. She had originally been treated for localised distal femoral osteosarcoma and had entered onto the 105AD7 study 21 weeks after completion of chemotherapy with cisplatin, doxorubicin and high-dose methotrexate. A routine follow-up chest radiograph performed at week 6 of the vaccine study (and 1 year from original diagnosis) had shown appearance of a single solitary 0.5 cm nodule, suspicious for metastasis. She completed the fourth dose of vaccine in the interval prior to excision of this nodule for diagnostic purposes, during which time the nodule decreased slightly on chest X-ray. At surgery, both this and a second, 2 mm nodule found in the other lung were excised completely and found to contain viable osteosarcoma. Despite the early relapse, the patient declined second line chemotherapy but was granted permission to continue a prolonged course of vaccination at 2 monthly intervals over a 2-year period. Immune response data (Figure 3B) showed a decrease in a pre-existing endogenous response, which was boosted after the third dose of vaccine. It is of note that at this timepoint the patients pulmonary metastasis stabilised. On continued vaccination, her *in vitro* immune response remained measurable except for a single sample at week 63. The patient remains free of recurrence 5.2 years from study entry and 3.2 years from last vaccination.

## DISCUSSION

105AD7 is a human anti-idiotypic antibody that binds to the monoclonal antibody 791T/36 and mimics the complement regulatory protein CD55. It has previously been shown to induce antitumour inflammatory responses that are associated with tumour cell apoptosis in colorectal cancer patients. As 791T/36 has been shown to stain osteosarcoma tumours and when radiolabelled has been used successfully in diagnostic imaging of these tumours. Therefore, osteosarcoma patients were potential candidates for 105AD7 vaccination. Colorectal cancer patients with minimal residual disease were shown to have better immune responses to 105AD7 than either patients with recurrent disease or patients with a large tumour burden (Durrant *et al*, 2000a; Maxwell-Armstrong *et al*, 2001). This may have been due to the immunosuppressive effect of a large tumour burden. The objectives of this study were therefore to assess the immune response to 105AD7 of children and young adults (<30 years of age) when commenced within 1–6 months of finishing systemic chemotherapy. Its main end points were toxicity and immune response, with evaluation of any clinical responses as a secondary end point.

There was no clinically significant toxicity associated with the vaccination even in the two patients who were given compassionate permission to continue being immunised for a 2-year period. This is in accord with the lack of significant toxicity seen in the colorectal cancer studies in older patients. 105AD7 combined i.d. and i.m. vaccine has now been administered safely to over 200 cancer patients across a wide age range.

The majority of patients (71%) showed an *in vitro* T-cell proliferation response to 105AD7 but not to the control human IgG. However, three immunisations were required to induce peak proliferative responses in the majority of patients. This is in contrast to the chemonaive colorectal cancer patients, who showed peak proliferation following their initial vaccination with 105AD7. Previous studies have shown that patients with an HLA-DR 1, 3 or 7 phenotype responded to 105AD7 vaccination. This observation was confirmed in this study with 80% of patients with these haplotypes showing a proliferation response to 105AD7. However, patients with a DR 13, 15 or 17 phenotype also responded suggesting that these haplotypes may also be able to present the class II peptide. This is not uncommon as many class II haplotypes have similar anchor residue requirements and show promiscuous binding of class II peptides (Chicz *et al*, 1993).

Lymphocytes responding to 105AD7 also secreted *γ*IFN suggesting that 105AD7 is inducing a Th1 immune response. This is in agreement with the low levels of anti-CD55 antibodies induced by this vaccine, suggesting that low-dose immunisation with 105AD7 predominantly induces a cellular Th1 rather than a Th2 response. This is in agreement with our animal studies that showed low doses of 105AD7 induced DTH responses whereas higher doses in combination with complete Freund's adjuvant induced anti-CD55 antibody responses (Austin *et al*, 1991).

Several patients had an apparent pre-existing proliferation response to 105AD7 prior to vaccination. In a recent randomised study in colorectal cancer, many of the unimmunised control patients showed an *in vitro* response to 105AD7 (unpublished results). An alternative suggestion is that disease regression associated with intensive chemotherapy induces an immune response to CD55 that can be detected *in vitro* with 105AD7. In this context six out of eight patients in group III also had an antibody response to CD55 prior to vaccination. High levels of CD55 released from dying osteosarcoma tumours presented in the context of inflammation may overcome immune ignorance or tolerance associated with this self-antigen. Further studies using antigen-specific ELISPOT assays will determine the frequency and specificity of these T-cell responses.

This trial was not designed to measure significant clinical benefit and only five patients with measurable disease were enrolled. However, disease status was followed in all patients pre- and postvaccination. Two patients showed evidence of clinical responses. One patient, who entered the study without measurable disease, had early lung metastasis, occurring within 1 year of original diagnosis, which was suspected during immunisation. She continued the vaccine on a compassionate basis for a total of 2 years without any other therapy. This patient remains disease free 4.7 years from time of metastasis. The second patient had chemorefractory primary disease that stabilised on 105AD7 immunisation. Immunisation was continued for a further 2 years and their disease has remained stable for a further 2 years since completion of vaccinations.

A theoretical concern in the design of this study was that the intensive chemotherapy used to treat osteosarcoma might prevent the mounting of a significant immune response when vaccination is commenced within a time frame of 6 months following end of chemotherapy, when patients are still considered immunosuppressed. This study demonstrates that this did not prevent this young group of patients responding to vaccination with 105AD7.

In conclusion, this study showed that 71% of young osteosarcoma patients were capable of mounting an immune response to an anti-idiotypic vaccine approach with 105AD7, commenced within a short time frame (6 months) of completion of intensive chemotherapy. There was no clinically significant toxicity associated with the vaccine. These results suggest that future trials of immunotherapy in osteosarcoma could include a vaccination strategy implemented early, when patients destined to relapse still have minimal disease burden. A randomised phase III study, with progression-free survival as an end point, is necessary to determine if this approach confers any survival benefit in osteosarcoma.

## Figures and Tables

**Table 1:** Immune responses of patients

		**Proliferation (SI)**			
**Patient**	**HLA/DR type**	**Pre**	**Post**	**Number of injections to observe response**	***γ*IFN (U ml^−1^)**
*Group I – Patients who failed to show a proliferative response to 105AD7 or human Ig*					
3	1, 3	1.3	1.6	—	0
6	13, 15	0.9	1.3	—	ND
12	1, 7	0.9	1.1	—	ND
21	13, 15	0.9	1.8	—	0
22^b^	11, 13	2.1	1.4	—	60
24^b^	4, 8	1.4	1.6	—	300
26	1, 7	1.5	1.1	—	0
28	7, 12	1.3	1.9	—	370

*Group II – Patients who showed a proliferative response to 105AD7 but not human Ig*					
20	7, 11	0.9	4.0	1	370
9^a^	4, 11	1.1	6.9	2	45
23	1, 7	1.1	3.1	2	0
31	ND	1.3	8.0	2	25
7	−17	1.3	19.6	4	0
16	4, 17	1.3	6.6	4	70
17	7, 17	1.1	2.1	4	ND

*Group III – Patients who had a pretreatment response to 105AD7 and who also showed a proliferative response to 105AD7 but not to human Ig*					
18^b^	12, 7	5.6	5.9	1	130
14	1, 4	3.9	6.4	2	0
15^b^	3, 4	2.4	6.2	2	ND
1^a^	1, 7	8.8	12.6	3	90
2	3, 7	6.4	7.4	3	185
8	1, 15	6.7	3.9	3	ND
10	1, 3	2.2	12.7	3	ND
27^b^	4, 13	8.5	19.9	3	60
5	−15	2.3	4.5	4	20
11	4, 13	9.6	4.6	4	0
13	−3	4.0	2.0	4	40
29	10, 17	2.0	2.0	4	0
4		2.5	7.5	1	0

a Patients received four doses of 105AD7 and compassionate immunisation quarterly for 2 years.

b Only three doses due to progressive disease.

^c^Patients were phenotyped for MHC class II using standard molecular techniques.

^d^Proliferation was assessed by 3H-labelled thymidine incorporation following 5-day stimulation with either 105AD7 or control human IgG.

^e^SI=cpm 105AD7/cpm hIgG.

^f^Secretion of *γ*IFN was assessed by sandwich ELISA (R&D Systems) following 5-day stimulation with 105AD7 or control human IgG. Only a few samples showed *γ*IFN secretion in response to human IgG and these values were considered background and were subtracted from the levels induced by 105AD7 stimulation.

**Table 2:** Clinical responses of patients

**Trial number**	**Clinical risk group**	**Interval from last chemo to vaccination**	**Immune response group**	**No. of prior relapses**	**Disease at study entry**	**Subsequent course**	**Current status**	**FU** a **(m)**	**Date of last FU info**
3	1	24	I	0	NMD	Never relapsed	ADF	45	09/04/02
21	1	16	I	2	NMD	Never relapsed	ADF	34	11/11/2000
26	1	21	I	0	NMD	Never relapsed	ADF	28	27/12/2001
28	2	25	I	0	NMD	Never relapsed	ADF	24	03/02/2003
6	1	14	I	2	NMD	Nodal and bone relapse	DOD	18	18/10/2001
12	1	12	I	0	NMD	Two further relapses	DOD	26	17/10/2000
22	1	22	I	2	Equivocal PD in lungs on CT	PD lungs	DOD	18	04/12/2002
24	1	12	I	3	Macroscopic residue	Local recurrence and lung mets	DOD	4	25/11/2002
9	1	22	II	0	Residual inoperable primary & metastatic disease	No further progressions	ADF	49	06/10/2003
17	1	22	II	1	NMD	No further relapse	ADF	33	26/06/2003
23	2	25	II	0	NMD	Never relapsed	ADF	31	20/07/2001
20	1	21	II	2	NMD	PD lungs	AWD	34	17/10/2002
7	1	20	II	1	Local recurrence	Local PD	DOD	6	27/01/2003
16	2	14	II	0	NMD	Two further relapses (lung and abdo)	DOD	19	20/11/2002
31	1	12	II	2	NMD	One further relapse	DOD	8	02/10/2001
1	2	21	III	0	NMD	pulm mets during vaccination	ADF	61	26/10/2003
5	1	27	III	1	NMD	No further relapse	ADF	45	02/01/03
10	1	25	III	1	NMD	Two skin relapses in thoracotomy scar	ADF	32	17/02/2000
11	2	8	III	0	NMD	Never relapsed	ADF	44	01/01/2003
29	2	18	III	0	NMD	Never relapsed	ADF	26	12/09/2001
2	1	19	III	3	NMD	Relapsed, Feb 2002	AWD	50	20/06/2002
4	1	11	III	2	NMD	Further pulm relapse	DOD	10	26/06/2003
8	1	11	III	0	NMD	Three further relapses	DOD	25	13/11/2002
13	1	9	III	1	NMD	One further relapse	DOD	36	24/05/2001
14	1	7	III	1	NMD	Further pulm relapse	DOD	17	13/02/2000
15	1	22	III	4	PD	Continued PD	DOD	2	22/06/2001
18	1	9	III	1	NMD	Local recurrence, then mediastinal recurrence, further local recurrence	DOD	16	07/07/2001
27	1	23	III	2	NMD	Pulmonary relapse	DOD	7	04/02/2003

NMD=no measurable disease; PD=progressive disease.

a FU is months from study entry.
